# Marine Viruses: Key Players in Marine Ecosystems

**DOI:** 10.3390/v9100302

**Published:** 2017-10-18

**Authors:** Mathias Middelboe, Corina P. D. Brussaard

**Affiliations:** 1 Marine Biological Section, University of Copenhagen, DK-3000 Helsingør, Denmark; 2 Department of Marine Microbiology and Biogeochemistry, NIOZ Royal Netherlands Institute of Sea Research, and University of Utrecht, P.O. Box 59, 1790 AB Den Burg, Texel, The Netherlands; corina.brussaard@nioz.nl

Viruses were recognized as the causative agents of fish diseases, such as infectious pancreatic necrosis and Oregon sockeye disease, in the early 1960s [1], and have since been shown to be responsible for diseases in all marine life from bacteria to protists, mollusks, crustaceans, fish and mammals [2]. However, it was not until the early 1990s that viral infections were discovered to affect marine systems beyond their role as pathogens of plants and animals, and viruses infecting unicellular organisms such as bacteria (i.e., the bacteriophages) and phytoplankton were shown to have a large influence on ecosystem processes. Since Karl-Heinz Moebus’ pioneering work on bacteriophage isolation and infection patterns obtained during a transect across the North Atlantic [3,4], research in marine viruses has developed into a significant and independent research field in marine biology, prompted by the increasing realization of the important and diverse roles of viruses in the marine ecosystem (e.g., [5,6]). The discovery that viruses were the most abundant biological entities in oceanic marine environments [7], reaching up to 10^8^ viruses mL^−1^, further stimulated marine virus research. Technical improvements in detection and enumeration of marine viruses (e.g., [8]) promoted advances in more detailed studies of viral abundance and diversity at high spatial and temporal resolution. Later, the expansion of virus research to coral reefs [9], sediments [10,11], the deep biosphere [12], and freshwater environments [13] emphasized that viruses are integrated inhabitants of all aquatic environments. Consequently, the past decades’ research has revealed viruses as key players in the marine ecosystem, from driving bacterial and algal mortality and evolution at the nanoscale, to influencing global-scale biogeochemical cycles and ocean productivity. The research has fundamentally changed our conceptual understanding of the function and regulation of aquatic ecosystems, and the development of molecular tools and DNA sequencing techniques has opened up for the exploration of viral diversity and the genetic mechanisms of virus-host interactions.

The present special issue aims at highlighting the progress in our understanding of the role of viruses and virus-host interactions in the marine environment by presenting novel research on the ecology, pathogenicity, distribution and diversity of marine viruses and the influence of virus-host interactions on mortality and evolution of marine microbial communities (Figure 1).

With the global increase in aquaculture, many of the viral pathogens have become severe causes of mortality in farmed organisms. Piscine orthoreovirus (PRV) is an example of a ubiquitous virus in sea water, which causes muscle inflammation in Atlantic salmon. In this special issue, Haatveit et al. [14] provide new insight on the infection kinetics of PRV, showing that the acute infection phase with high virus production is followed by reduced transcription of viral RNA, and the virus is maintained in the fish at a low persistent level. Pathogens of marine animals, however, constitute a very small fraction of marine viruses, as the majority of viruses infect bacteria and protists. Recent studies have shown that, even though viruses are typically 10-fold more abundant than bacteria in marine surface water, there are large variations in the virus-bacteria ratio across marine environments [15]. By examining the influence of environmental conditions on the relationship between viruses and bacteria using multivariate models, Finke et al. [16], here, demonstrate that environmental factors, such as inorganic nutrient concentrations, are important predictors of host and, consequently, viral abundance—and thus virus-host ratios—across a broad range of temporal and spatial scales. Similarly, trophic interactions in the microbial food web, such as predation and the availability of limiting nutrients, were shown to affect the structure and function of viral and prokaryote communities [17].

A three-year study on the virus-host dynamics of haptophyte phytoplankton and their dsDNA viruses showed seasonal fluctuations in specific virus populations indicating shifts in viral communities in response to seasonal variations in host diversity [18]. At the same time, the presence of persistent viral genotypes throughout the study period suggested co-existence between specific viruses and their hosts. Understanding of the environmental drivers of virus-host interaction is essential for predicting how environmental changes will affect virus-driven processes in a future ocean [19]. Highfield et al. [20] showed that elevated pCO_2_ levels can affect the composition and diversity of *Emiliania huxleyi* viruses (EhV). Maat et al. [21] found highly specific temperature sensitivity in virus infectivity and production for four newly isolated viruses infecting the Arctic picophytoplankter *Micromonas polaris*. As the predicted warming of the Arctic regions will stimulate *Micromonas* growth rates and promote growth earlier in the season, the authors suggest that viral production will likely do the same.

The increased accumulation of viral metagenomic data the past decade has revealed a huge viral diversity (e.g., [22,23]), and the marine virome is considered the largest pool of unexplored genetic diversity on the globe, with 63–93% of the sequences not represented in the public databases [24]. A recent analysis of viral metagenomic sequence data from 43 surface ocean sites identified ~5500 populations of dsDNA viruses, of which only 39 could be affiliated to cultured viruses [22]. Even at very small scales, viral diversity may be high, as demonstrated by Flaviani et al. [25], who found 254 unique virus phylotypes in a 250 mL oceanic water sample, supporting previous suggestions that local viral diversity is relatively similar to global diversity [22].

The large number of unknown viral populations in the marine metagenome emphasizes the need for further isolation, characterization and sequencing of specific marine viruses. This special issue presents several new marine viruses of eukaryotes (*Prymnesium parvum*, [26]) and bacteria (*Shewanella*, [27], *Vibrio anguillarum* [28] and *Dinoroseobacter shibae* [29]), adding to the rapidly growing database of genome-sequenced and characterized marine viruses. Several auxiliary metabolic genes and other functional genes were identified in the phage genomes, suggesting a mutual benefit for both phage and host that could potentially be disseminated to other hosts by horizontal gene transfer (Figure 1). Prophage-encode genes can thus contribute to host functional properties, including virulence, by so-called lysogenic conversion, potentially expanding the niches occupied by the lysogenized hosts (Figure 1). Further, prophage induction can stimulate biofilm formation by promoting the release of extracellular DNA, which becomes a component in the biofilm matrix [30]. The paper by Leigh et al. [27] shows that lytic phage infections also enhance biofilm formation in *Shewanella*, which forms biofilms in the gut of the tunicate *Ciona intestinalis*. *Shewanella* is part of a complex relationship between the *C. intestinalis* and its associated microbiome, and the study demonstrates that phage interaction with its *Shewanella* host contributes to the symbiotic relationships between the gut microbiome and the tunicate host (Figure 1).

Viruses may also acquire accessory genes from their eukaryotic or prokaryotic hosts [31] (Figure 1). By expressing these genes during infection, the viruses may augment key steps in cellular metabolism and ultimately increase virus production [32]. In addition to using viral genes acquired from the host, viruses may also control the expression of host genes during infection to promote viral production or inhibit host defense systems. This was demonstrated by Fedida & Lindell [33], where expression patterns of specific host genes in the cyanobacterium *Synechococcus* sp strain WH8102 during cyanophage infection suggested that the phage exploited the host genes for improved infection efficiency.

The high local viral diversity obtained from oceanic metagenomic data [22] suggests a high dispersal of viral genes across the sampled ocean viral communities. Moebus [4] had already demonstrated that bacterial viruses with specific infectious properties were distributed across large spatial scales in the North Atlantic. Later, a worldwide distribution of a virus infecting the picophytoplankton *Micromonas pusilla* was reported by Cottrell and Suttle [34], suggesting that viruses are efficiently spread in the marine environment. This is supported by the study by Kalatzis et al. [28], which demonstrates that H20-like vibriophages infecting the fish pathogen *Vibrio anguillarum* are globally distributed either as free phages or as prophages inside bacterial genomes. The authors argue that selection for co-existence, rather than arms race dynamics, might explain the global distribution of near-identical H20-like bacteriophages and their prevalence as prophages in *Vibrio* genomes.

Viral host cells have developed multiple defense strategies against lytic viral infections (Figure 1). These include both mutational changes in the cell surface receptors providing resistance to phage adsorption, and various mechanisms for destroying the viral DNA upon infection (e.g., restriction modification and CRISPR-Cas defense) [35]. Mordecai et al. [36] propose a different life cycle strategy of the dsDNA EhV viruses infecting the coccolithophorid *Emiliania huxleyi*, where the detection of viral RNA in the virus-resistant haploid cell of *E. huxelyi* suggested a new mechanism of infection, and the co-existence of viruses and host. Defense strategies are often associated with a fitness cost, as surface modification mutations may have an influence on, e.g., substrate uptake or enzyme secretion [37], and because virus inactivation mechanisms may be expensive to maintain [38]. Such trade-offs between resistance and fitness costs were explored in the two groups of eukaryotic phytoplankton, *Ostreococcus tauri* [39], and *E. huxleyi* [40]. Surprisingly, no direct cost of resistance was detected in these systems, emphasizing the complexity of interplay between virus-host co-evolution and the environmental conditions.

The large and diverse group of nucleocytoplasmic large DNA viruses (NCLDV) includes a number of viral families infecting small photosynthetic protists, thus affecting mortality, evolution and production of these phytoplankton. In the current special issue, the research on NCLDV infecting phytoplankton is represented by the Prasinoviruses infecting the ubiquitous group of pico-sized Prasinophycea such as *Micromonas* and *Ostreococcus* [21,39,41], and viruses infecting bloom-forming haptophytes such as *Prymnesium parvum* [18,26] and *Emiliania huxleyi* [20,31,36,40]. These studies highlight the progression in our understanding of the role of viruses infecting eukaryotic algae, provide a synthesis of the current knowledge in the field, and identify gaps in our knowledge surrounding viral life history and interactions with their hosts.

The discovery of the giant *Acanthamoeba polyphaga* mimivirus stimulated a new line of research, exploring the ecology and evolution of the group of large DNA viruses infecting eukaryotic protists, including the haptophyte *Phaeocystis globosa* [42]. Here, Wilhelm et al. [43] synthesize the current knowledge and common characteristics of this group of novel viruses and their interactions with their hosts, as well as their virophage parasites.

The compilation of papers included in the current special issue highlights the exploration of eukaryotic and prokaryotic viruses, from discovery to complex interplays between virus and host and the interactions with ecologically relevant environmental variables. The discovery of novel viruses and new mechanisms underlying virus distribution and diversity exemplify the fascinating world of marine viruses. The oceans greatly shape Earth’s climate, hold 1.37 billion km^3^ of seawater, produce half the half of the oxygen in the atmosphere, and are integral to all known life. In a time where life in the oceans is under increasing threat (global warming, acidification, pollution, economic use), it is pressing to understand how viruses affect host population dynamics, biodiversity, biogeochemical cycling and ecosystem efficiency.

## Figures and Tables

**Figure 1:**
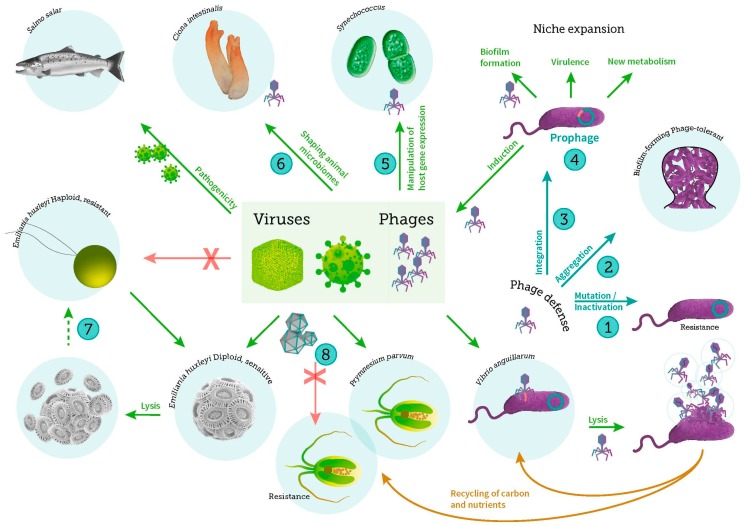
Schematic overview of important virus-host interactions in the marine ecosystem covered in this special issue, including viral infection of bacteria, phytoplankton, and fish. Specific explanations of key interactions: (1) Bacteria can prevent phage infection by mutational modification of surface receptors or by enzymatic degradation of the incoming phage DNA; (2) Alternatively, protection of cells in aggregates or biofilms can be a defense strategy against phage infection; (3) Infection by temperate phages can result in the integration of the phage DNA in the host genome as prophage; The integrated prophage can prevent infection by similar phages (Superinfection exclusion mechanism) and (4) contribute with important genetic information to the host that may expand its metabolic or virulence properties. Prophage induction leads to the release of new phages and may also stimulate biofilm formation; (5) Phages can manipulate host gene expression in cyanobacteria for improved infection efficiency, either by exploiting the host genes or by encoding host photosynthesis genes which are then expressed during infection; (6) Phage interaction with their bacterial hosts contributes to shaping the gut microbiome of invertebrates (e.g., tunicates), thus affecting the symbiotic relationship between gut microbes and their hosts; (7) In the coccolithophorid phytoplankton *Emiliania huxleyi* the diploid virally infected cells may undergo viral induced lysis or re-emerge (dotted arrow) as haploid cells containing viral RNA and lipids. These haploid cells are thought to resist virus infection (as indicated with the X) and develop into the diploid cells by karyogamy; (8) The large and diverse group of nucleocytoplasmic large DNA viruses (NCLDV) infects a range of photosynthetic protists such as the prasinophytes *Micromonas pusilla* and *Ostreococcus tauri* and, as exemplified in the figure, the toxin-producing haptophyte *Prymnesium parvum*, thus affecting mortality, diversity and production of phytoplankton. These interactions are strongly controlled by environmental factors such as temperature, nutrient availability and light. As for bacteria, several mechanisms of resistance (indicated with an X) to viruses have been described in the photosynthetic protists (see text for further details).
